# Exploring the In Vitro Antibacterial Properties of *Milicia regia* and *Entandrophragma angolensis*: Insight Into Their Antibiofilm and Efflux Pump Inhibitory Activities

**DOI:** 10.1155/tswj/2641156

**Published:** 2026-06-19

**Authors:** Samuel Korsah, Michael Ofori, Michelle Opoku Aboagyewaah, Kakraba Geoffrey, Michael Osei Boateng, Jessica Korsah, Miriam Tagoe, Theophilus Ninkyi, Cynthia Amaning Danquah

**Affiliations:** ^1^ Department of Biology, University of New Brunswick, Fredericton, Canada, unb.ca; ^2^ Department of Pharmaceutical Sciences, School of Pharmacy, Central University, Accra, Ghana, central.edu.gh; ^3^ Department of Pharmaceutical Sciences, Wa Technical University, Wa, Upper West Region, Ghana; ^4^ Department of Pharmacology, Kwame Nkrumah University of Science and Technology, Kumasi, Ghana, knust.edu.gh

**Keywords:** antimicrobial resistance, biofilm, efflux pump, *Entandrophragma angolensis*, extracts, HT-SPOTi, microorganisms, *Milicia regia*, phytomedicines

## Abstract

**Introduction:**

Biofilms are breeding grounds for adapted and acquired antibiotic resistance through increased efflux activities and horizontal gene transfer. Medicinal plants are sources of antimicrobial agents for the treatment of bacterial, parasitic, and fungal infections.

**Aim:**

In this research, we examined antimicrobial, antibiofilm, and efflux pump inhibition activity of the methanolic extracts of the stem barks of *Milicia regia* and *Entandrophragma angolensis*.

**Methods:**

Crude methanolic extracts were assessed using three distinct assays: the high‐throughput spot culture growth inhibition (HT‐SPOTi) assay for bacterial growth inhibition, a crystal violet–based antibiofilm screening assay to quantify their biofilm‑inhibitory activity and the ethidium bromide accumulation assay for evaluating changes in bacterial cell membrane permeability against *Mycobacterium smegmatis*, *Mycobacterium aurum*, *Staphylococcus aureus*, and *Pseudomonas aeruginosa*.

**Results:**

The preliminary qualitative phytochemical screening suggested the presence of tannins, flavonoids, terpenoids, glycosides, alkaloids, and saponins. The minimum inhibitory concentrations for extracts against *S*. *aureus, P*. *aeruginosa, M*. *aurum*, and *M*. *smegmatis* were 250, 125, 500, and 250 *μ*g/mL, respectively, and for *E*. *angolensis:* 125, 125, 500, and 500 *μ*g/mL, respectively. Both plants displayed significant (∗∗∗*ρ* < 0.005) biofilm inhibition activities against all bacteria with the highest inhibition recorded in *S*. *aureus*: *M*. *regia*, *E*. *angolensis*, and the reference drug ciprofloxacin were 73%, 62%, and 79%, respectively.

**Conclusion:**

The extracts produced marked antiefflux pump effects against *S.aureus* and *P. aeruginosa*. This study established the antibacterial, antibiofilm, and efflux pump inhibitory capacities of *M*. *regia* and *E*. *angolensis* and provides the rationale for their folkloric uses in the treatment of infections.

## 1. Introduction

Bacterial pathogens have been a significant threat to humanity, resulting in numerous lethal infectious diseases [1, 2]. Bacterial infections have shaped some of the most challenging periods in human history [3]. The most significant of these were the “three pandemic plagues,” each caused by *Yersinia pestis*, an extraordinarily virulent gram‐negative bacterium [4, 5]. These pandemics resulted in millions of fatalities in a brief span, profoundly reshaping human societies. The 20th‐century discovery of antibiotics, whether bactericidal or bacteriostatic, revolutionized the management of bacterial infections [6, 7]. Antibiotics enabled the treatment of previously incurable diseases by reducing bacterial growth and, as a result, infection. Although decades of antibiotic use have dramatically reduced the morbidity and mortality associated with bacterial infections, the global health burden imposed by these pathogens remains substantial [8, 9]. Bacterial infections, for example, accounted for 13.6% of all deaths worldwide in 2019 [10]. Five pathogens were responsible for 54% of all bacterial‐related fatalities: the gram‐positive organisms *Staphylococcus aureus* and *Streptococcus pneumoniae*, and the gram‐negative species *Escherichia coli*, *Klebsiella pneumoniae*, and *Pseudomonas aeruginosa*. Decades of misuse and overuse of antibiotics have accelerated the evolution of drug‐resistant variants, contributing directly to the escalating crisis of antimicrobial resistance (AMR).

The rise in AMR is quietly stripping away the advances of modern medicine, exposing humanity to the specter of uncontrollable infections [11]. The spread of multidrug resistant (MDR) and extensively‐drug resistant (XDR) bacteria has become a major public health concern, having a devastating impact on the most vulnerable individuals, including those with weakened immune systems and critically ill patients in intensive care, resulting in dire clinical consequences and increased risk of fatal outcomes [12, 13]. Through the activation of bacterial efflux pumps and the formation of biofilms, bacteria have devised sophisticated strategies to resist the therapeutic effects of antibiotics, rendering previously effective antibiotics ineffective against the onslaught of infectious diseases [14, 15].

Biofilms are complex dynamic communities of multiple microbial species encased in a self‐produced three‐dimensional matrix, composed of extracellular polymeric substances (EPS) [16, 17]. This protective matrix creates a secure synergistic environment often referred to as “safe haven,” where microorganisms can thrive, resist antimicrobial agents, and evade the host′s immune responses, ultimately promoting their survival and persistence [18]. Biofilms create a powerful shield around bacteria, making them up to 1000 times more resistant to antibiotics [19]. This shield is present in 75% of pathogenic bacteria, making treatment very difficult. Cells within biofilms exhibit a remarkable ability to irreversibly bind to diverse surfaces, including living tissues and medical devices like catheters, valves, and prosthetics [20, 21]. This strong attachment enables the establishment of chronic, resilient colonies, leading to persistent infections and device‐related problems posing significant treatment challenges [22]. Novel strategies are required to overcome the cells′ strong grip and eradicate the biofilm, as untreated biofilms can have severe consequences such as chronic infections, device failure, and compromised patient outcomes. Microbial biofilms are a key factor in making infections more severe, increasing the cost of treatment and prolonging hospital stays [23, 24]. Additionally, research has shown that certain types of bacteria known as tolerant and persister bacteria play a significant role in the persistence and recurrence of biofilm‐related infections, making them harder to treat. Effective management and innovative treatments are essential to combat the persistence of biofilm‐associated cells [25, 26].

Another major mechanism that contributes immensely to bacterial evolution is the expression of bacterial efflux pumps. Efflux pumps are bacterial membrane transporters that facilitate the active translocation of substrates such as antibiotics, dyes, metabolites, quorum‐sensing signals, and virulence factors [27]. Efflux pumps are proteins present in the cell membranes of all living organisms, including bacteria and human cells [28]. Efflux pumps play a crucial role in antibiotic resistance by removing antibiotics from bacterial cells, reducing their effectiveness [29]. They help export toxins produced by the cell, maintaining cellular homeostasis. Commonly, MDR bacteria possess multiple efflux pumps to expel antimicrobial agents. Efflux pumps eradicate harmful substances such as heavy metals from the bacterial cells and also contribute to the formation of biofilms, resulting in AMR [30]. Recent investigations indicate that various elements can trigger the efflux pumps and promote the emergence of MDR pathogens, including environmental cues, regulatory proteins, and numerous mutations linked to efflux pump–associated genes [31, 32]. Environmental signals such as lincomycin and boric acid have been observed to activate efflux pumps and lead to a temporary decrease in susceptibility to antibiotics in *Stenotrophomonas maltophilia* [33, 34].

Efflux pump inhibitors (EPIs) and biofilm‐forming inhibitors can potentiate antibiotic activity by disrupting efflux systems that normally expel antibiotics from bacterial cells, thereby increasing intracellular drug accumulation[31, 35]. EPIs can specifically interfere with the operation of efflux pumps, inhibiting the removal of antibiotics and resulting in increased vulnerability of bacteria to a range of antibiotics. Thus, the use of EPIs may be a potential way to control MDR bacterial infections.

The secondary metabolites present in medicinal plants have shown potential as therapeutic agents, offering a natural source for the development of new treatments and medicines [36]. These compounds, produced by plants for their own defense and survival, can be leveraged to create effective solutions for various health issues [37]. Medicinal plants contain a wide range of compounds with diverse structures and mechanisms, offering a rich source for developing novel treatments for drug‐resistant infections [38]. According to Blanco et al. (2016) safe use in many communities, these plants have yielded compounds like quinones, phenols, alkaloids, flavonoids, and terpenoids which have demonstrated potent antimicrobial activity against resistant pathogens. The traditional approach to antibiotic discovery involved screening soil microbes like actinobacteria for their ability to suppress harmful bacterial growth [28]. This was highly successful in the past, leading to the discovery of iconic antibiotics like penicillin and streptomycin. However, this approach became less effective due to overreliance on familiar sources and neglect of how new antibiotics can overcome bacterial defenses [39]. As a result, researchers are now seeking innovative strategies to combat the escalating issue of antibiotic resistance [26].

In this study, two native African medicinal plants *Milicia regia* and *Entandrophragma angolensis*, known for their ethnomedicinal uses such as treatment of boil and fever, were investigated to find out their potential to inhibit bacteria and possess some varying mechanisms to tackle AMR.

*E*. *angolensis*, also called African mahogany or Edinam by the Akan′s in Ghana, is a valued hardwood tree found in many parts of Africa. This remarkable tree can reach heights of up to 60 m, with trunk diameters that can be as wide as 1.5 m. Its heartwood has a beautiful light pinkish‐brown color that darkens as it matures, whereas the sapwood retains a lighter tone. Known for its strength and durability, African mahogany is also resistant to termites and decay caused by fungi. The traditional medicinal uses of *E. angolensis* are well‐recognized, as it has been employed to treat wounds, burns, and a range of health conditions like menstrual cramps and digestive issues [39]. The plant′s therapeutic potential is attributed to its wide variety of natural compounds, which include alkaloids, flavonoids, polyphenols, saponins, and tannins. These compounds are celebrated for their strong antioxidants, anti‐inflammatory, and antimicrobial effects, suggesting that *E. angolensis* holds significant promise for future medical applications [40, 41].

On the other hand, *M*. *regia*, also referred to as Odum by the Akan in Ghana, belonging to the Moraceae family, is native to West Africa, spanning from Senegal and Gambia to Ghana. This species can achieve heights of over 47 m and diameters of 2.5 m, demonstrating remarkable resilience and adaptability in its environment. The wood quality is noted for its strength, durability, and resistance to pest and fungal attacks, making it highly valued in the timber industry [42]. Recent laboratory studies on *M. regia* revealed that its leaf extracts exhibit potent anti‐inflammatory properties through the inhibition of key inflammatory mediators, making it a candidate for further exploration in inflammatory disorders [43]. Additionally, the phytochemical analysis of the bark revealed the presence of secondary metabolites with cytotoxic effects against various cancer cell lines, indicating potential applications in cancer therapies. This work seeks to investigate the in vitro antibacterial, antibiofilm, and EPI activities of *M*. *regia* and *E*. *angolensis*.

## 2. Materials and Methods

### 2.1. Materials

The materials used in this study were as follows: test tubes, beakers, heating mantles, separating funnel, filter paper, funnel, dropping pipette, measuring cylinder, 96‐well half‐skirted PCR plates, 96‐well microtiter plates (Starlab, United Kingdom), Tryptase soy broth (Himedia laboratory), fluorimeter (Jos. Hansen, Germany), and spectrophotometer (Jos. Hansen, Germany).

### 2.2. Chemicals and Reagents

We performed the experiments using the following chemicals and reagents: Dragendoff′s reagent, ferric chloride, dilute NaOH, chloroform, concentrated sulfuric acid (H_2_SO4), Ethanol (LabChem), ethidium bromide (EtBr) solution (Sigma‐Aldrich Inc), OADC, 0.5% (*V*/*V*) glycerol, 0.2% (*V*/*V*) Tween 80, verapamil (Ernest Chemist Ltd, Ghana), 80% glucose *w*/*v* (Sigma G7528).

### 2.3. Plant Collection and Processing

**Figure 1:**
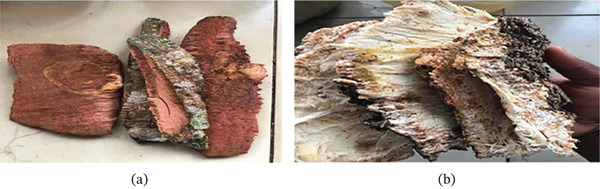
Stem bark of plants: (a) *Milicia regia* and (b) *Entandrophragma angolensis.*

**Figure 2:**
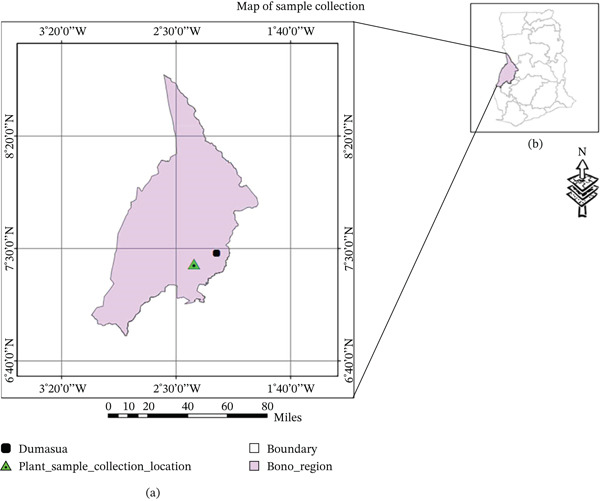
(a) Map of Ghana showing the collection area (Dumasua) in (b) mauve purple highlighting the Bono region in mauve purple and a detailed map of the plant collection area. The map was generated using ArcMap Desktop10.7.1.

The stem barks of *E*. *angolensis* and *M*. *regia* (Figure 1) were collected from Dumesua, BY‐0344‐2736 Sunyani west municipality (Figure 2) in the Bono region of Ghana in February 2024. The plant samples were identified and authenticated by Miss Miriam Tagoe, the head of Herbarium at the Department of Pharmaceutical Sciences, School of Pharmacy, Central University, with the Voucher Numbers CUC/B/KU/010 and CUC/B/KU/009, respectively. The barks of *M*. *regia* were washed under running water, cut into smaller chunks, sun‐dried for a month, and coarsely milled. About 800 g of the powdered stem was extracted using 3 L of methanol 98% using the cold maceration method. The plant extract was filtered and oven‐dried at 40°C to yield a dark brown crude extract. The dried extracts were stored at 4°C until further use.

Yield %=mass of extractmass of plant material×100

### 2.4. Phytochemical Screening

A preliminary qualitative phytochemical analysis was performed to identify key plant secondary metabolites in the stem barks of *E*. *angolensis* and *M*. *regia*, including tannins, saponins, flavonoids, terpenoids, glycosides, alkaloids, and coumarins. Standard protocols were followed to qualitatively identify the presence of these compounds, providing insight into the plant′s chemical composition and potential therapeutic properties. The various testing protocols used were as prescribed by Trease and Evans [44].

#### 2.4.1. Test for Tannins

Three drops of FeCl_2_ were added to the 1‐mL filtrate of the stem barks of *E*. *angolensis* and *M*. *regia;* formation of dark green color indicates the presence of condensed tannins, whereas the formation of dark blue color indicates the presence of hydrolysable tannins.

#### 2.4.2. Test for Saponins

On 1 mL of filtrate of the stem barks of *E*. *angolensis* and *M*. *regia*, 2 mL of distilled water was added and shaken vigorously. The mixture was allowed to stand for 10 min, and the formation of foam on the surface of the mixture persisting more than 10 min indicated the presence of saponins.

#### 2.4.3. Test for Flavonoids

One milliliter of filtrate of the stem barks of *E*. *angolensis* and *M*. *regia* was added to 2 mL of dilute NaOH. A golden yellow color indicated the presence of flavonoids.

#### 2.4.4. Test for Glycosides

A total of 2 mL of the extract of the stem barks of *E*. *angolensis* and *M*. *regia*, 3 mL of chloroform, and 10% ammonia solution were added. Formation of a pink color indicates the presence of glycosides.

#### 2.4.5. Test for Terpenoids

A mixture of 5 mL of extract of the stem barks of *E*. *angolensis* and *M*. *regia* and 2 mL of chloroform were formed; four drops of concentrated sulfuric acid were carefully added. A reddish brown layer at the junction indicated the presence of terpenoids.

#### 2.4.6. Test for Alkaloids

Four drops of 2% H_2_SO_4_ were added to 5 mL of extract of the stem barks of *E*. *angolensis* and *M*. *regia*, filtered, and three drops of Dragendoff′s reagent added to 1 mL of filtrate. Orange red precipitation suggested the presence of alkaloids.

#### 2.4.7. Test for Coumarins

Two milligrams of the extracts of the stem barks of *E*. *angolensis* and *M*. *regia* were dissolved in 2 mL of methanol and 10% NaOH was added dropwise. A yellow precipitate indicated the presence of coumarins.

### 2.5. In Vitro Antibacterial Screening

The antibacterial activity of the crude extracts were assessed using the high‐throughput spot culture growth inhibition (HT‐SPOTi) assay [27, 45]. In this screening, Middlebrook 7H10 (MB7H10) agar was used to culture *Mycobacterium* strains such as *Mycobacterium smegmatis* (NCTC 8159) and *Mycobacterium aurum* (NCTC 10437), whereas nutrient agar was used in culturing *S*. *aureus* (ATCC 25923) and *P*. *aeruginosa* (ATCC 27853). A two‐fold serial dilution of the extracts was performed in 0.3% dimethyl sulfoxide (DMSO) using PCR half‐skirted 96‐well plates to obtain a broad concentration range. Bacterial strains were inoculated into 10 mL of molten agar contained in Falcon tubes and incubated at 37°C for 18–24 h. Following incubation, microbial growth on the agar surface was harvested by washing with 0.9% (*w*/*v*) normal saline solution. Serial dilution was initiated by adding 100 *μ*L of the washed bacterial suspension to a Falcon tube containing 10 mL of sterile normal saline. Subsequently, 1 mL of this mixture was transferred to a second Falcon tube containing 9 mL of sterile normal saline. A further 1‐mL aliquot from this second tube was transferred into a third Falcon tube containing 19 mL of sterile normal saline, completing the serial dilution procedure. The contents of the final dilution tube were used for the HT‐SPOTi assay. Additionally, 2 *μ*L each of E. *angolensis* and M*. regia* extracts from the PCR half‐skirted 96‐well plate were dispensed into the designated wells of a 96‐well microtiter plate as previously described [46].

To each well of the plate containing the predispensed *E*. *angolensis* and *M. regia*, 200 *μ*L of the prepared molten agar was added, followed by the addition of 2 *μ*L of the bacterial suspension (from the final Falcon tube) using a multichannel pipette. The plate was then covered, sealed with parafilm, and wrapped in aluminum foil to prevent contamination and light exposure. Incubation was carried out at 37°C for 18–24 h. Following incubation, the plates were examined, and the minimum inhibitory concentration (MIC) was determined.

### 2.6. Biofilm Inhibition Assay

The method used for determining the antibiofilm activity of natural agents was adapted from previously described protocols [47–49]. Quantitative assessment of biofilm formation and inhibition was performed using a 96‐well microtiter plate assay [48]. *M*. *smegmatis* (NCTC 8159), *M*. *aurum* (NCTC 10437), *S*. *aureus* (ATCC 25923), and *P*. *aeruginosa* (ATCC 27853) were each inoculated into 3–5 mL of sterile trypticase soy broth (TSB) and incubated at 37°C for 24 h. After incubation, bacterial cultures were diluted 1:100 in sterile TSB. One hundred microliters (100 *μ*L) of the diluted bacterial suspension were dispensed into designated wells of a sterile 96‐well microtiter plate. The experimental design included two wells per extract, one well for the standard drug control (ciprofloxacin), one negative control (bacterial cells only), and two blank controls (broth plus extract only). An additional 100 *μ*L of 5% (*w*/*v*) extract solution was added to the appropriate wells containing the bacterial suspension. Plates were then covered with cling film, wrapped in aluminum foil to limit light exposure, and incubated at 37°C for 24 h to allow biofilm formation. Following incubation, well contents were aspirated, and each well was thoroughly washed with sterile phosphate‐buffered saline (PBS) to remove planktonic cells. Biofilms were stained by adding 125 *μ*L of 0.1% (*w*/*v*) crystal violet solution to each designated well and incubating for 10 min at room temperature. Excess stains were removed by washing with distilled water, and plates were air‐dried. To solubilize the bound crystal violet, 200 *μ*L of 95% ethanol was added to each well, followed by incubation at room temperature for 15 min. Finally, 125 *μ*L of the ethanol–crystal violet solution from each well was transferred in triplicate to a separate 96‐well plate for absorbance measurement.

Percentage biofilm inhibition=absorbance of control−absorbance of testabsorbance of control ×100

where the control is bacterial cells only, and blank is the growth media and drug only.

### 2.7. EtBr Accumulation Assay

A streamlined assay employing EtBr was used to evaluate the EPI activity of test compounds [27, 50]. EtBr is a well‐characterized efflux pump substrate. This protocol represents an improved and optimized version of existing methods. Verapamil (125 mg/L) was used as the reference EPI for comparison. *M*. *smegmatis* (NCTC 8159), *M*. *aurum* (NCTC 10437), *S*. *aureus* (ATCC 25923), and *P*. *aeruginosa* (ATCC 27853) were cultured in Middlebrook 7H9 (MB7H9) and nutrient broth, respectively, enriched with albumin, dextrose, and catalase (ADC supplement). Cultures were grown to an optical density (OD) of 0.8. To prepare the working inoculum, 5 mL of bacterial suspension was mixed with 5 mL of fresh MB7H9 broth and nutrient broth, and the OD was adjusted to 0.4 using additional broth or culture as necessary in sterile Falcon tubes. The bacterial suspension at OD 0.4 was centrifuged at 3000 rpm for 10 min. After discarding the supernatant, the bacterial pellet was resuspended in 10 mL of sterile PBS by vortexing to ensure uniform mixing. The MICs of the extracts, determined previously, were reduced by half to obtain subinhibitory concentrations for use in the assay.

For the test samples, 500 *μ*L of the buffered bacterial suspension was added to labeled Eppendorf tubes. For the blank controls, 500 *μ*L of sterile PBS was used. To each tube (test and blank), 2 *μ*L of the sub‐MIC test extract was added along with 2.5 *μ*L of 80% (*w*/*v*) glucose solution. The contents were mixed thoroughly by vortexing. Aliquots of 100 *μ*L from each mixture were transferred to wells of a sterile 96‐well microtiter plate. Then, 5 *μ*L of EtBr solution (50 mg/L) was added to each well just before measurement.

Fluorescence readings were taken immediately using a fluorimeter set to an excitation wavelength of 530 nm and an emission wavelength of 600 nm. Relative fluorescence units (RFUs) were recorded over time, and the data were analyzed by plotting RFU versus time to evaluate efflux activity and the inhibitory effects of the test compounds.

### 2.8. Statistical Analysis

Results expressed as mean ± standard error of mean (SEM). Utilizing GraphPad Software Prism 9.0, statistical analysis and graphing were performed (Graph pad Software Inc., San Diego, California, United States). Significant differences between groups were determined using one‐way analysis of variance (ANOVA) followed by Tukey′s multiple comparisons.

## 3. Results and Discussion

The stem barks of *E*. *angolensis* and *M*. *regia had a percentage yield* of 7.2% and 5.3%, respectively.

### 3.1. Phytochemical Screening of the Stem Bark of *M*. *regia* and *E*. *angolensis*

Qualitative phytochemical screening revealed that methanolic extracts of *M*. *regia* (MRE) and *E*. *angolensis* (EAE) contain glycosides, tannins, alkaloids, triterpenoids, saponins, and flavonoids (Table 1).

**Table 1:** Preliminary qualitative phytochemical screening of extracts.

Phytoconstituents	*Milicia regia*	*Entandrophragma angolensis*
Tannins	Detected	Detected
Saponins	Detected	Detected
Glycosides	Detected	Detected
Terpenoids	Detected	Detected
Coumarin	Not detected	Not detected
Alkaloids	Detected	Detected
Flavonoids	Detected	Detected

### 3.2. Antibacterial Activity of *M*. *regia* and *E*. *angolensis* Bark Extracts

The crude extracts showed comparable antibacterial effects against both gram‐positive and gram‐negative bacterial strains in the HT‐SPOTi assay. The MICs for both extracts against *S*. *aureus, P*. *aeruginosa, M*. *aurum*, and *M*. *smegmatis* showed some levels of inhibitory effects, with a greater effect seen in *S*. *aureus* and *P*. *aeruginosa* (Table 2).

**Table 2:** Minimum inhibitory concentration (MIC) of *Milicia regia and Entandrophragma angolensis* (*μ*g/mL).

Microorganisms	*Milicia regia*	*Entandrophragma angolensis*	Ciprofloxacin	Isoniazid
*S. aureus*	250	125	10.7	—
*P. aeruginosa*	125	125	0.625	—
*M. aurum*	500	500	—	62.5
*M. smegmatis*	250	500	—	62.5

### 3.3. Biofilm Inhibitory Effects of *M*. *regia* and *E*. *angolensis* Bark Extracts

**Figure 3:**
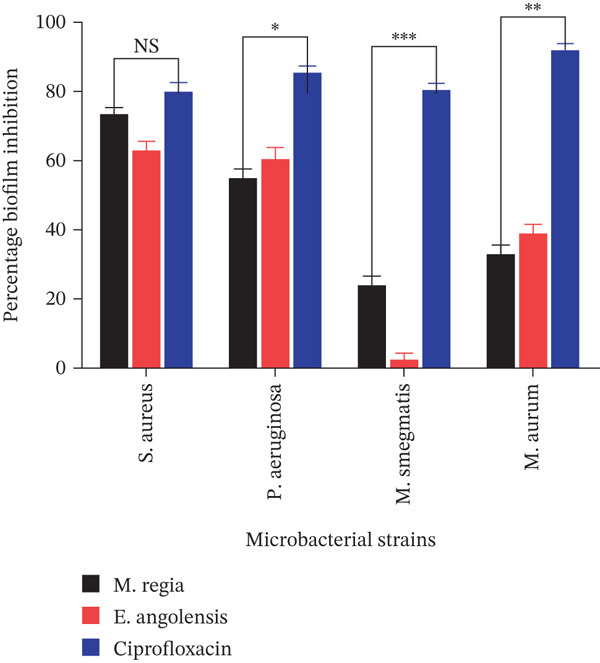
Antibiofilm formation activity of crude extracts and standard (ciprofloxacin) in *S. aureus, P. aeruginosa, M. aurum,* and *M. smegmatis.* Data were expressed as mean ± SEM. Two‐way analysis of variance followed by Tukey′s multiple comparison test. ####*ρ* < 0.005 of ciprofloxacin versus *M. regia* and *E. angolensis.* *ρ* > 0.05 of ciprofloxacin versus *M. regia* and *E. angolensis* against *S. aureus,* ∗*ρ* < 0.05 of ciprofloxacin versus *M. regia* and *E. angolensis* against *P. aeruginosa.* ∗∗∗*ρ* < 0.005 of ciprofloxacin versus *M. regia* and *E. angolensis* against *M. aurum.* ∗∗*ρ* < 0.05 of ciprofloxacin versus *M. regia* and *E. angolensis* against *M. smegmatis.* Ciprofloxacin is a standard biofilm inhibitor and was compared with *M. regia* and *E. angolensis.*

Crude extracts obtained from the stem bark of *M*. *regia* and *E*. *angolensis* were evaluated for their ability to inhibit biofilm formation by *S*. *aureus*, *P*. *aeruginosa*, *M*. *aurum*, and *M*. *smegmatis*. The percentage inhibition of biofilm formation for all tested samples is presented in Figure 3. For both extracts, the antibiofilm activity did not display a concentration‐dependent pattern. Against *S. aureus*, both extracts exhibited moderate inhibitory effects, with *M*. *regia* showing the highest activity. The observed biofilm inhibition percentages for *M*. *regia*, *E*. *angolensis*, and the reference drug ciprofloxacin were 73 %, 62%, and 79%, respectively. In contrast, both extracts showed biofilm inhibition against *P. aeruginosa*, *M. smegmatis*, and *M. aurum*, with *M. smegmatis* exhibiting the lowest inhibition levels overall. Specifically, the biofilm inhibition percentages against *P. aeruginosa* were 30% for *M*. *regia* and 54% for *E*. *angolensis*. Against *M. smegmatis*, inhibition was 23% for *M*. *regia* and 2% for *E*. *angolensis*. For *M. aurum*, biofilm inhibition was measured at 32% and 38% for *M*. *regia* and *E*. *angolensis*, respectively.

### 3.4. EPI Activity of *M*. *regia* and *E*. *angolensis* Bark Extracts

The potential EPI activity of the crude extracts were evaluated using an EtBr accumulation assay. EtBr accumulation profiles were monitored over 60 min for *M*. *aurum, S*. *aureus, P*. *aeruginosa*, and *M*. *smegmatis* in the presence of the crude extracts. These results were compared with those obtained using verapamil, a known standard EPI. For all the bacterial species, the extracts from *M*. *regia* and *E*. *angolensis* exhibited markedly some level of EPI activity compared with verapamil.

## 4. Discussion

The management of infectious diseases is increasingly challenged by the rise of MDR and XDR pathogens, representing a major global public health threat [13, 51, 52]. The efficacy of existing antibiotics is declining due to widespread drug resistance within healthcare systems, underscoring the urgent need to identify and develop new compounds with novel mechanisms of action for treating infectious diseases. This resistance crisis has also resulted in prolonged treatment durations, which can lead to poor patient adherence and subtherapeutic dosing [53, 54]. As AMR continues to undermine the effectiveness of current treatment regimens, the development of new therapeutic options is critical to address this growing threat.

Natural products, particularly medicinal plants, represent a valuable source for the discovery of novel anti‐infective agents [55, 56]. Numerous plant species have been studied for their therapeutic potential against various infectious diseases. Recent investigations have highlighted the antibacterial activity of several indigenous Ghanaian plants traditionally employed in the treatment of respiratory ailments, skin infections, wound healing, and other conditions [55, 57, 58].

The aim of this study was to investigate some common medicinal plants in Ghanaian traditional medicine, such as *M*. *regia* and *E*. *angolensis* extracts for their anti‐infective properties and explore their effects on some mechanisms that contribute to AMR. To facilitate the process to identify potential leads in the drug discovery process, extracts were screened for their secondary metabolites as prescribed by Treas and Evans Pharmacognosy. This is a proven technique in the natural products drug discovery field, which informed the identification of active compounds from different natural product sources, including plants.

Plants possess a wider range of secondary metabolites, and the presence of these metabolites represents the medicinal properties of plants [59–61]. The qualitative screening suggested *M. regia and E*. *angolensis* extracts contain glycosides, tannins, alkaloids, triterpenoids, saponins, and flavonoids (Table 1). The bioactivities exhibited by the extracts could be due to these suggested secondary metabolites present in the plants. However, these findings are preliminary and do not constitute a definitive compound identification. Further chromatographic and spectroscopic analysis on the plants would be necessary to achieve definitive confirmation and identification of the compounds.

The antimicrobial activities of *M. regia* and *E*. *angolensis* were determined using an investigative technique known as HT‐SPOTi assay [62, 63]. The HT‐SPOTi method is a variation of the agar dilution technique and was used to assess the antibacterial activity of the extracts against microorganisms. It is designed to screen multiple samples simultaneously in a relatively quick and efficient manner, making it suitable for high‐throughput applications [46]. The results in Table 2 showed *M. regia* and *E*. *angolensis* produced some level of inhibition against *S. aureus, P. aeruginosa, M. aurum*, and *M. smegmatis. M*. *regia* extract produced an MIC of 250, 125, 500, and 250 *μ*g/mL, respectively, against *S. aureus, P. aeruginosa, M. aurum*, *and M. smegmatis.* On the other hand, the MIC values for *E*. *angolensis* against *S. aureus, P. aeruginosa, M. aurum, and M. smegmatis* were recorded to be 125, 125, 500, and 500 *μ*g/mL, respectively. The MIC values obtained suggested the antibacterial activities of *M. regia* and *E*. *angolensis* extracts.

The expression of efflux pumps encoded within bacterial genomes is a major contributor to AMR [14, 35, 64]. These pumps actively expel antimicrobial agents from the bacterial cell, resulting in subtherapeutic intracellular drug concentrations [64]. This mechanism can drive adaptive evolutionary responses in bacteria, enabling them to better survive in challenging environments. The underlying principle of this assay relies on the use of EtBr, a well‐characterized efflux pump substrate that fluoresces when accumulated within living microbial cells but remains nonfluorescent outside the cells. The level of fluorescence is directly related to the intracellular accumulation of EtBr. Fluorescence measurements were obtained using a fluorimeter and plotted as a function of time.

**Figure 4:**
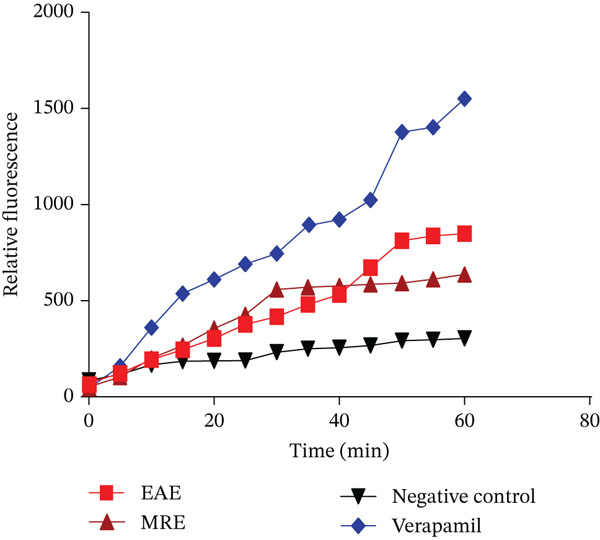
Antiefflux pump effects of *Milicia regia* and *Entandrophragma angolensis* extracts against *Staphylococcus aureus.*

**Figure 5:**
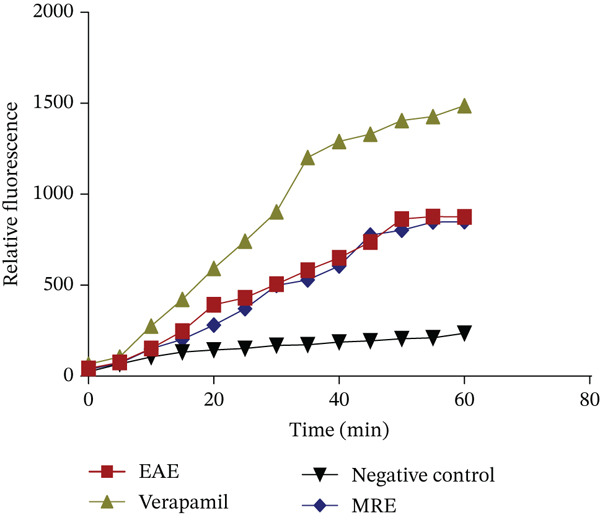
Antiefflux pump effects of *Milicia regia* and *Entandrophragma angolensis* extracts against *Pseudomonas aeruginosa.*

**Figure 6:**
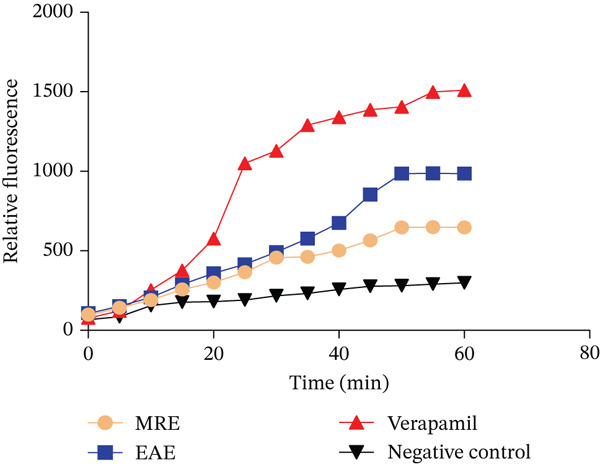
Antiefflux pump effects of *Milicia regia* and *Entandrophragma angolensis* extract against *Mycobacterium aurum.*

**Figure 7:**
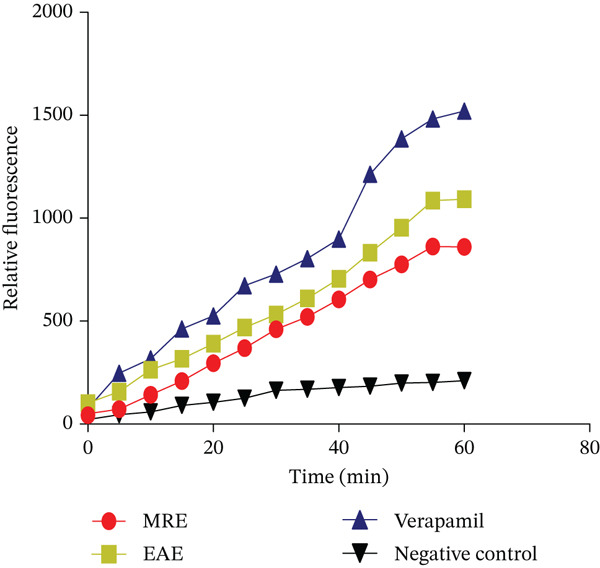
Antiefflux pump effects of *Milicia regia* and *Entandrophragma angolensis* extract against *Mycobacterium smegmatis.*

The extracts of *E*. *angolensis* and *M. regia* produced marked antiefflux pump effects against *S. aureus and P. aeruginosa* when compared with verapamil, which was used as a standard EPI (Figures 4 and 5). The antiefflux pump effect of *E*. *angolensis* and *M. regia* was determined against surrogate model species of *Mycobacterium tuberculosis* and was shown to have produced good inhibitory effects on the *Mycobacterium* efflux pumps (Figures 6 and 7). It has been reported that agents exhibiting antiefflux pump activity can enhance the susceptibility of microorganisms to therapeutic drugs by mitigating the development of drug resistance [35]. EPIs act synergistically with antibiotics by blocking efflux mechanisms, thereby maintaining elevated intracellular antibiotic concentrations [62, 65]. By disrupting the efflux pump function, EPIs prevent the active expulsion of antibiotics, resulting in increased bacterial sensitivity to these agents. Consequently, the use of EPIs represents a promising strategy for managing MDR bacterial infections.

The ability of plant extracts to exhibit antibiofilm activity and EPI is highly dependent on the solvents used during extraction, as different solvents selectively extract specific classes of bioactive compounds while excluding others. Biofilms, which are structured communities of bacteria encased in an extracellular matrix, and efflux pumps, which actively expel antimicrobial agents from bacterial cells, both play crucial roles in antimicrobial [63, 66]. Certain plant‐derived compounds, such as polyphenols, flavonoids, alkaloids, and terpenoids, have been shown to disrupt biofilm formation or inhibit efflux pumps.

In this work, the extracts of *E*. *angolensis* and *M. regia* were able to significantly (∗∗∗*ρ* < 0.005) reduce the formation of bacterial biofilm in all the studied microorganisms such as *S. aureus, P. aeruginosa, M. aurum, and M. smegmatis.* The effects of *E*. *angolensis* and *M. regia* were significant (∗∗∗*ρ* < 0.005) when compared with the ciprofloxacin treatment group against *S. aureus and P. aeruginosa,* as presented in Figure 3 and Table 3. With their percentage biofilm inhibitory effects, *E*. *angolensis* and *M. regia* produced percentage inhibitory effects of 63% and 75%, respectively, against *S. aureus* compared with 79% recorded for ciprofloxacin. Against *P. aeruginosa, E*. *angolensis* and *M. regia* produced 60% and 55% percentage inhibition, respectively, with 82% for ciprofloxacin. The level of inhibition of *E*. *angolensis* and *M. regia* against the *Mycobacterium* strains such as *M. aurum and M. smegmatis* was moderately achieved, with percentage inhibition of 10% and 22% against *M. smegmatis,* 40% and 35% against *M. aurum,* compared with ciprofloxacin as shown in Figure 3 and Table 3. However, agents that function as both EPIs and biofilm inhibitors may exert their effects through multiple mechanisms, potentially reducing the likelihood of resistance development. Accordingly, *E*. *angolensis* and *M. regia* may represent promising candidates for further investigation as dual‐action antimicrobial agents that could reverse AMR.

**Table 3:** Percentage biofilm inhibitions of crude extracts.

Samples	% inhibition in *S. aureus*	% inhibition in *P. aeruginosa*	% inhibition in *M. smegmatis*	% inhibition in *M. aurum*
*Milicia regia*	73 ± 2.32	54 ± 2.21	23 ± 1.92	32 ± 1.38
*Entandrophragma angolensis*	62 ± 1.78	59 ± 1.44	2 ± 1.51	38 ± 2.44
Ciprofloxacin	79 ± 1.04	87 ± 3.07	82 ± 2.31	91 ± 2.82

*Note:* Data were expressed as mean ± SEM with Dunnett′s multiple comparison test.

## 5. Conclusion

The results indicate that the examined plants extracts may serve as promising antimicrobial and antibiofilm agents, providing an alternative strategy to address AMR. Additional studies are required to assess their potential use in the development of new adjuvant therapies.

## Funding

No funding was received for this manuscript.

## Ethics Statement

The stem barks of *Entandrophagma angolensis* (EAE) and *Milicia regia* (MRE) were collected from Dumesua, Sunyani west municipality in the Bono region of Ghana. The plant samples were identified and authenticated by Miss Miriam Tagoe, the head of Herbarium at the Department of Pharmaceutical Sciences, School of Pharmacy, Central University with the Voucher Numbers CUC/B/KU/010 and CUC/B/KU/009, respectively. Therefore, no license or authorization is needed to gather plant material for this research work.

## Consent

The authors have nothing to report.

## Conflicts of Interest

The authors declare no conflicts of interest.

## Data Availability

The datasets generated during and/or analyzed during the current study are available from the corresponding author on request.

## References

[1] Soni J. , Sinha S. , and Pandey R. , Understanding Bacterial Pathogenicity: A Closer Look at the Journey of Harmful Microbes, Frontiers in Microbiology. (2024) 15, 1370818, 10.3389/fmicb.2024.1370818, 38444801. 38444801 PMC10912505

[2] Ristori M. V. , Guarrasi V. , Soda P. , Petrosillo N. , Gurrieri F. , Longo U. G. , Ciccozzi M. , Riva E. , and Angeletti S. , Emerging Microorganisms and Infectious Diseases: One Health Approach for Health Shared Vision, Genes. (2024) 15, no. 7, 908, 10.3390/genes15070908, 39062687. 39062687 PMC11275270

[3] Souza P. F. N. , Filho N. S. S. , Mororó J. L. T. , Brito D. M. S. , da Lima A. B. , Mesquita F. P. , and Montenegro R. C. , Pandemic Events Caused by Bacteria Throughout Human History and the Risks of Antimicrobial Resistance Today, Microorganisms. (2025) 13, no. 2, 457, 10.3390/microorganisms13020457, 40005822. 40005822 PMC11858245

[4] Sati H. , Carrara E. , Savoldi A. , Hansen P. , Garlasco J. , Campagnaro E. , Boccia S. , Castillo-Polo J. A. , Magrini E. , Garcia-Vello P. , Wool E. , Gigante V. , Duffy E. , Cassini A. , Huttner B. , Pardo P. R. , Naghavi M. , Mirzayev F. , Zignol M. , Cameron A. , Tacconelli E. , Aboderin A. , al Ghoribi M. , al-Salman J. , Amir A. , Apisarnthanarak A. , Blaser M. , el-Sharif A. , Essack S. , Harbarth S. , Huang X. , Kapoor G. , Knight G. , Muhwa J. C. , Monnet D. L. , Ousassa T. , Sacsaquispe R. , Severin J. , Sugai M. , Taneja N. , and Umubyeyi Nyaruhirira A. , The WHO Bacterial Priority Pathogens List 2024: A Prioritisation Study to Guide Research, Development, and Public Health Strategies Against Antimicrobial Resistance, Lancet Infectious Diseases. (2025) 25, no. 9, 1033–1043, 10.1016/S1473-3099(25)00118-5, 40245910. 40245910 PMC12367593

[5] Moyo P. , Bodede O. , Wooding M. , Famuyide I. M. , Makhubu F. N. , Khorommbi N. K. , Ofori M. , Danquah C. A. , McGaw L. J. , and Maharaj V. J. , Quorum Sensing Inhibition by South African Medicinal Plants Species: An In Vitro and An Untargeted Metabolomics Study, BMC Complementary Medicine and Therapies. (2025) 25, no. 1, 138, 10.1186/s12906-025-04880-4, 40221765. 40221765 PMC11994000

[6] Hutchings M. I. , Truman A. W. , and Wilkinson B. , Antibiotics: Past, Present and Future, Current Opinion in Microbiology. (2019) 51, 72–80, 10.1016/j.mib.2019.10.008. 31733401

[7] Muteeb G. , Rehman M. T. , Shahwan M. , and Aatif M. , Origin of Antibiotics and Antibiotic Resistance, and Their Impacts on Drug Development: A Narrative Review, Pharmaceuticals. (2023) 16, no. 11, 1615, 10.3390/ph16111615, 38004480. 38004480 PMC10675245

[8] Salam M. A. , al-Amin M. Y. , Salam M. T. , Pawar J. S. , Akhter N. , Rabaan A. A. , and Alqumber M. A. A. , Antimicrobial Resistance: A Growing Serious Threat for Global Public Health, Healthcare, 2023, 11, no. 13, 1946, 10.3390/healthcare11131946, 37444780. 37444780 PMC10340576

[9] Zhang C. , Fu X. , Liu Y. , Zhao H. , and Wang G. , Burden of Infectious Diseases and Bacterial Antimicrobial Resistance in China: A Systematic Analysis for the Global Burden of Disease Study 2019, Lancet Regional Health–Western Pacific. (2024) 43, 100972, 10.1016/j.lanwpc.2023.100972. 38076321 PMC10700598

[10] Diop M. , Guitoula C. , Tamouh A. G. , Youbong T. , Daffé S. M. M. , Ndoye M. , Gueye M. W. , Wone F. , Ngom M. , Seck M. , Youm N. , Bassoum O. , Lakhe N. A. , Ba P. S. , Faye A. , and Gning S. B. , Prevalence of Bacterial Infections and Factors Associated With Death Related to These Infections in Two Medical Departments of a Tertiary Hospital in Dakar, Senegal, IJID regions. (2025) 15, 100623, 10.1016/j.ijregi.2025.100623, 40230497. 40230497 PMC11994947

[11] Tse Sum Bui B. , Auroy T. , and Haupt K. , Fighting Antibiotic-Resistant Bacteria: Promising Strategies Orchestrated by Molecularly Imprinted Polymers, Angewandte Chemie International Edition. (2022) 61, no. 8, e202106493, 10.1002/anie.202106493, 34779567. 34779567

[12] Marino A. , Maniaci A. , Lentini M. , Ronsivalle S. , Nunnari G. , Cocuzza S. , Parisi F. M. , Cacopardo B. , Lavalle S. , and la Via L. , The Global Burden of Multidrug-Resistant Bacteria, Epidemiologia. (2025) 6, no. 2, 21, 10.3390/epidemiologia6020021, 40407562. 40407562 PMC12101290

[13] Almutairy B. , Extensively and Multidrug-Resistant Bacterial Strains: Case Studies of Antibiotics Resistance, Frontiers in Microbiology. (2024) 15. 10.3389/fmicb.2024.1381511 PMC11256239 39027098

[14] Gaurav A. , Bakht P. , Saini M. , Pandey S. , and Pathania R. , Role of Bacterial Efflux Pumps in Antibiotic Resistance, Virulence, and Strategies to Discover Novel Efflux Pump Inhibitors, Microbiology. (2023) 169, no. 5, 001333, 10.1099/mic.0.001333. 37224055 PMC10268834

[15] Abbas S. H. , Kiani H. S. , Gohar F. , Zahra S. , Javed A. , Khan S. , and Khan D. , Understanding the Role of Bacterial Biofilm in Antibiotic Resistance: Defensive Strategies and Clinical Challenges, Exploring Bacterial Biofilms, 2025, IntechOpen, 10.5772/intechopen.1009440.

[16] Patra S. , Saha S. , Singh R. , Tomar N. , and Gulati P. , Biofilm Battleground: Unveiling the Hidden Challenges, Current Approaches and Future Perspectives in Combating Biofilm Associated Bacterial Infections, Microbial Pathogenesis. (2025) 198, 107155, 10.1016/j.micpath.2024.107155. 39586337

[17] Damyanova T. and Paunova-Krasteva T. , What We Still Don′t Know About Biofilms—Current Overview and Key Research Information, Microbiology Research. (2025) 16, no. 2, 46, 10.3390/microbiolres16020046.

[18] Liu X. , Yao H. , Zhao X. , and Ge C. , Biofilm Formation and Control of Foodborne Pathogenic Bacteria, Molecules. (2023) 28, no. 6, 2432, 10.3390/molecules28062432, 36985403. 36985403 PMC10058477

[19] Römling U. and Balsalobre C. , Biofilm Infections, Their Resilience to Therapy and Innovative Treatment Strategies, Journal of Internal Medicine. (2012) 272, no. 6, 541–561, 10.1111/joim.12004, 23025745. 23025745

[20] Khatoon Z. , McTiernan C. D. , Suuronen E. J. , Mah T. F. , and Alarcon E. I. , Bacterial Biofilm Formation on Implantable Devices and Approaches to Its Treatment and Prevention, Heliyon. (2018) 4, no. 12, e01067, 10.1016/j.heliyon.2018.e01067, 30619958. 30619958 PMC6312881

[21] Geremia N. , Giovagnorio F. , Colpani A. , de Vito A. , Botan A. , Stroffolini G. , Toc D. A. , Zerbato V. , Principe L. , Madeddu G. , Luzzati R. , Parisi S. G. , and di Bella S. , Fluoroquinolones and Biofilm: A Narrative Review, Pharmaceuticals. (2024) 17, no. 12, 10.3390/ph17121673, 39770514. PMC11679785 39770514

[22] Bouhrour N. , Nibbering P. H. , and Bendali F. , Medical Device-Associated Biofilm Infections and Multidrug-Resistant Pathogens, Pathogens. (2024) 13, no. 5, 393, 10.3390/pathogens13050393, 38787246. 38787246 PMC11124157

[23] Sahoo K. , Meshram S. , and Sahoo K. , Biofilm Formation in Chronic Infections: A Comprehensive Review of Pathogenesis, Clinical Implications, and Novel Therapeutic Approaches, Cureus. (2024) 16, no. 10, 10.7759/cureus.70629, e70629, 39483571. 39483571 PMC11527504

[24] Zafer M. M. , Mohamed G. A. , Ibrahim S. R. M. , Ghosh S. , Bornman C. , and Elfaky M. A. , Biofilm-Mediated Infections by Multidrug-Resistant Microbes: A Comprehensive Exploration and Forward Perspectives, Archives of Microbiology. (2024) 206, no. 3, 101, 10.1007/s00203-023-03826-z, 38353831. 38353831 PMC10867068

[25] Sharma S. , Barman P. , Joshi S. , Preet S. , and Saini A. , Multidrug Resistance Crisis During COVID-19 Pandemic: Role of Anti-Microbial Peptides as Next-Generation Therapeutics, Colloids and Surfaces B: Biointerfaces. (2022) 211, 112303, 10.1016/j.colsurfb.2021.112303, 34952285. 34952285 PMC8685351

[26] Sedighi O. , Bednarke B. , Sherriff H. , and Doiron A. L. , Nanoparticle-Based Strategies for Managing Biofilm Infections in Wounds: A Comprehensive Review, ACS Omega. (2024) 9, no. 26, 27853–27871, 10.1021/acsomega.4c02343, 38973924. 38973924 PMC11223148

[27] Ofori M. , Danquah C. A. , Ativui S. , Doe P. , and Asamoah W. A. , In-Vitro Anti-Tuberculosis, Anti-Efflux Pumps and Anti-Biofilm Effects of Crinum asiaticum Bulbs, Biomedical and Pharmacology Journal. (2021) 14, no. 4, 1905–1915, 10.13005/bpj/2289.

[28] Blanco P. , Hernando-Amado S. , Reales-Calderon J. , Corona F. , Lira F. , Alcalde-Rico M. , Bernardini A. , Sanchez M. , and Martinez J. , Bacterial Multidrug Efflux Pumps: Much More Than Antibiotic Resistance Determinants, Microorganisms. (2016) 4, no. 1, 14, 10.3390/microorganisms4010014, 27681908. 27681908 PMC5029519

[29] Mishra R. , Panda A. K. , de Mandal S. , Shakeel M. , Bisht S. S. , and Khan J. , Natural Anti-Biofilm Agents: Strategies to Control Biofilm-Forming Pathogens, Frontiers in Microbiology. (2020) 11, 566325, 10.3389/fmicb.2020.566325, 33193155. 33193155 PMC7658412

[30] Roca I. , Espinal P. , Vila-Farrés X. , and Vila J. , The Acinetobacter baumannii Oxymoron: Commensal Hospital Dweller Turned Pan-Drug-Resistant Menace, Frontiers in Microbiology. (2012) 3, 148, 10.3389/fmicb.2012.00148, 22536199. 22536199 PMC3333477

[31] Zhang S. , Wang J. , and Ahn J. , Advances in the Discovery of Efflux Pump Inhibitors as Novel Potentiators to Control Antimicrobial-Resistant Pathogens, Antibiotics. (2023) 12, no. 9, 1417, 10.3390/antibiotics12091417, 37760714. 37760714 PMC10525980

[32] Vareschi S. , Jaut V. , Vijay S. , Allen R. J. , and Schreiber F. , Antimicrobial Efflux and Biofilms: An Interplay Leading to Emergent Resistance Evolution, Trends in Microbiology. (2025) 33, no. 9, 1018–1032, 10.1016/j.tim.2025.04.012, 40410028. 40410028

[33] Zając O. M. , Tyski S. , and Laudy A. E. , Phenotypic and Molecular Characteristics of the MDR Efflux Pump Gene-Carrying Stenotrophomonas maltophilia Strains Isolated in Warsaw, Poland, Biology. (2022) 11, no. 1, 105, 10.3390/biology11010105, 35053103. 35053103 PMC8772754

[34] Klimkaitė L. , Drevinskaitė R. , Krinickis K. , Sužiedėlienė E. , and Armalytė J. , Stenotrophomonas maltophilia of Clinical Origin Display Higher Temperature Tolerance Comparing With Environmental Isolates, Virulence. (2025) 16, no. 1, 2498669, 10.1080/21505594.2025.2498669, 40314203. 40314203 PMC12064055

[35] Ren J. , Wang M. , Zhou W. , and Liu Z. , Efflux Pumps as Potential Targets for Biofilm Inhibition, Frontiers in Microbiology. (2024) 15, 1315238, 10.3389/fmicb.2024.1315238, 38596384. 38596384 PMC11002903

[36] Santos-Aberturas J. and Vior N. M. , Beyond Soil-Dwelling Actinobacteria: Fantastic Antibiotics and Where to Find Them, Antibiotics. (2022) 11, no. 2, 195, 10.3390/antibiotics11020195, 35203798. 35203798 PMC8868522

[37] Demain A. L. , Importance of Microbial Natural Products and the Need to Revitalize Their Discovery, Journal of Industrial Microbiology and Biotechnology. (2014) 41, no. 2, 185–201, 10.1007/s10295-013-1325-z, 23990168. 23990168

[38] Shakya A. K. , Medicinal Plants: Future Source of New Drugs, International Journal of Herbal Medicine. (2016) 4, no. 4, 59–64.

[39] Vaou N. , Stavropoulou E. , Voidarou C. , Tsigalou C. , and Bezirtzoglou E. , Towards Advances in Medicinal Plant Antimicrobial Activity: A Review Study on Challenges and Future Perspectives, Microorganisms. (2021) 9, no. 10, 2041, 10.3390/microorganisms9102041, 34683362. 34683362 PMC8541629

[40] Pradubyat N. , Wunnakup T. , Praparatana R. , Wongwiwatthananukit S. , Jongrungruangchok S. , Songsak T. , Madaka F. , and Sudsai T. , Evaluation of Antioxidant and Anti-Inflammatory Properties, Bioactive Compound Profiling, and Molecular Mechanisms of a Multicomponent Thai Herbal Formulation, Phytomedicine Plus. (2024) 4, no. 4, 100662, 10.1016/j.phyplu.2024.100662.

[41] Daoud G. , Ahsan F. , Mahmood T. , Bano S. , Ansari V. A. , Zaidi S. M. H. , and Ansari J. A. , Therapeutic Potential and Bioactive Compounds of Apium graveolens: A Phytopharmacological Review, Pharmacological Research-Reports. (2025) 3, 100039, 10.1016/j.prerep.2025.100039.

[42] Ofori D. and Cobbinah J. , Integrated Approach for Conservation and Management of Genetic Resources of Milicia Species in West Africa, Forest Ecology and Management. (2007) 238, no. 1-3, 1–6, 10.1016/j.foreco.2006.09.091.

[43] Shewale V. D. , Deshmukh T. A. , Patil L. S. , and Patil V. R. , Anti-Inflammatory Activity ofDelonix regia (Boj. Ex. Hook), Advances in Pharmacological and Pharmaceutical Sciences. (2012) 2012, no. 1, 789713, 10.1155/2012/789713. PMC3206388 22110490

[44] Evans W. C. , Trease and Evans′ Pharmacognosy, Elsevier Health Sciences, 2009.

[45] Moyo P. , Ofori M. , Bodede O. S. , Wooding M. , Khorommbi N. K. , McGaw L. J. , Danquah C. A. , and Maharaj V. J. , Investigation of the Antimycobacterial Activity of African Medicinal Plants Combined With Chemometric Analysis to Identify Potential Leads, Scientific Reports. (2024) 14, no. 1, 14660, 10.1038/s41598-024-65369-7, 38918410. 38918410 PMC11199645

[46] Danquah C. A. , Maitra A. , Gibbons S. , Faull J. , and Bhakta S. , HT-SPOTi: A Rapid Drug Susceptibility Test (DST) to Evaluate Antibiotic Resistance Profiles and Novel Chemicals for Anti-Infective Drug Discovery, Current Protocols in Microbiology. (2016) 40, no. 1, 17.8.1, 10.1002/9780471729259.mc1708s40, 26855282. 26855282

[47] Iaconis A. , de Plano L. M. , Caccamo A. , Franco D. , and Conoci S. , Anti-Biofilm Strategies: A Focused Review on Innovative Approaches, Microorganisms. (2024) 12, no. 4, 639, 10.3390/microorganisms12040639, 38674584. 38674584 PMC11052202

[48] Kırmusaoğlu S. , Biofilm and Screening Antibiofilm Activity of Agents, 2019, Antibiotic Resistance, Antibiofilm Strategies and Activity Methods, Antimicrobials, 99, 10.5772/intechopen.78751.

[49] Mastoor S. , Nazim F. , Rizwan-ul-Hasan S. , Ahmed K. , Khan S. , Ali S. N. , and Abidi S. H. , Analysis of the Antimicrobial and Anti-Biofilm Activity of Natural Compounds and Their Analogues Against Staphylococcus aureus Isolates, Molecules. (2022) 27, no. 20, 6874, 10.3390/molecules27206874, 36296467. 36296467 PMC9610881

[50] Al-Sallami D. , Alsultan A. , Abbas K. , and Clarke S. , Evaluation of Efflux Pump Inhibitory Activity of Some Plant Extracts and Using Them as Adjuvants to Potentiate the Inhibitory Activity of Some Antibiotics Against Staphylococcus aureus, Open Veterinary Journal. (2023) 13, no. 1, 42–47, 10.5455/OVJ.2023.v13.i1.5, 36777436. 36777436 PMC9897506

[51] Almakrami M. , Salmen M. , Aldashel Y. A. , Alyami M. H. , Alquraishah N. , AlZureea M. , and Almakrami J. , Prevalence of Multidrug-, Extensively Drug-, and Pandrug-Resistant Bacteria in Clinical Isolates From King Khaled Hospital, Najran, Saudi Arabia, Discover Medicine. (2024) 1, no. 1, 108, 10.1007/s44337-024-00094-8.

[52] Rastmanesh S. , Zeinaly I. , Alivirdiloo V. , Mobed A. , and Darvishi M. , Biosensing for Rapid Detection of MDR, XDR and PDR Bacteria, Clinica Chimica Acta. (2025) 567, 120121, 10.1016/j.cca.2024.120121, 39746435. 39746435

[53] Muteeb G. et al., Antimicrobial Resistance: Linking Molecular Mechanisms to Public Health Impact. SLAS, Discovery. (2025) 100232. 10.1016/j.slasd.2025.100232 40216324

[54] Gajic I. , Tomic N. , Lukovic B. , Jovicevic M. , Kekic D. , Petrovic M. , Jankovic M. , Trudic A. , Mitic Culafic D. , Milenkovic M. , and Opavski N. , A Comprehensive Overview of Antibacterial Agents for Combating Multidrug-Resistant Bacteria: The Current Landscape, Development, Future Opportunities, and Challenges, Antibiotics. (2025) 14, no. 3, 221, 10.3390/antibiotics14030221, 40149033. 40149033 PMC11939824

[55] Ofori M. , Danquah C. A. , Ossei P. P. S. , Rahamani G. , Asamoah W. A. , Ativui S. , and Doe P. , Acute and Sub-Acute Toxicity Studies of the Chloroform Extract of Crinum asiaticum Bulbs in Mice, South African Journal of Botany. (2021) 143, 133–140, 10.1016/j.sajb.2021.07.047.

[56] Nortey N. N. D. , Korsah S. , Tagoe M. , Apenteng J. A. , Owusu F. A. , Oppong J. , Attah A. E. , and Allotey S. , Herbs Used in Antimalarial Medicines: A Study in the Greater Accra Region of Ghana, Evidence-Based Complementary and Alternative Medicine. (2023) 2023, no. 1, 6697078, 10.1155/2023/6697078, 37636997. 37636997 PMC10460277

[57] Quartey A. K. , Korsah S. , Apenteng J. A. , Nortey N. N. D. , Tagoe M. , Mintah D. N. , Barfour A. F. , Owusu F. W. A. , and Kontoh D. B. , A Study of the Antimicrobial and Wound Healing Activities of the Ethanolic Leaf Extract of Anchomanes difformis (Blume) Engl. Pallidus, Pallidus. Journal of Advances in Medical and Pharmaceutical Sciences. (2024) 26, no. 5, 66–75, 10.9734/jamps/2024/v26i5688.

[58] Ofori M. , Danquah C. A. , Asante J. , Ativui S. , Doe P. , Abdul-Nasir Taribabu A. , Nugbemado I. N. , and Mensah A. N. , Betulin and Crinum asiaticum L. Bulbs Extract Attenuate Pulmonary Fibrosis by Down Regulating Pro-Fibrotic and Pro-Inflammatory Cytokines in Bleomycin-Induced Fibrosis Mice Model, Heliyon. (2023) 9, no. 6, e16914, 10.1016/j.heliyon.2023.e16914, 37346329. 37346329 PMC10279834

[59] Roy A. , Khan A. , Ahmad I. , Alghamdi S. , Rajab B. S. , Babalghith A. O. , Alshahrani M. Y. , Islam S. , and Islam M. R. , Flavonoids a Bioactive Compound From Medicinal Plants and Its Therapeutic Applications, BioMed Research International. (2022) 2022, no. 1, 5445291, 10.1155/2022/5445291, 35707379. 35707379 PMC9192232

[60] Wink M. , Modes of Action of Herbal Medicines and Plant Secondary Metabolites, Medicines. (2015) 2, no. 3, 251–286, 10.3390/medicines2030251, 28930211. 28930211 PMC5456217

[61] Korsah S. , Gbedema S. Y. , Bayor M. T. , Boakye-Gyasi M. E. , Owusu F. W. A. , and Forkuo A. D. , In Vivo Antimalarial Activity of Polyalthia longifolia (Annonaceae) Leaf Extract and Assessment of Its Formulated Oral Dosage Forms, Evidence-Based Complementary and Alternative Medicine. (2021) 2021, no. 1, 8, 10.1155/2021/6519346. PMC8632403 34858510

[62] Zack K. M. , Sorenson T. , and Joshi S. G. , Types and Mechanisms of Efflux Pump Systems and the Potential of Efflux Pump Inhibitors in the Restoration of Antimicrobial Susceptibility, With a Special Reference to Acinetobacter baumannii, Pathogens. (2024) 13, no. 3, 197, 10.3390/pathogens13030197, 38535540. 38535540 PMC10974122

[63] Ahmed Z. T. , Singh P. , Saraswathi A. , Deekshitha S. , Kumar R. R. S. , Thacharodi A. , and Hassan S. , Introduction to Microbial Biofilms, Omics Approaches in Biofilm Research: Perspectives and Applications, 2025, Springer, 3–43, 10.1007/978-3-031-91863-6_1.

[64] Kumawat M. , Nabi B. , Daswani M. , Viquar I. , Pal N. , Sharma P. , Tiwari S. , Sarma D. K. , Shubham S. , and Kumar M. , Role of Bacterial Efflux Pump Proteins in Antibiotic Resistance Across Microbial Species, Microbial Pathogenesis. (2023) 181, 106182, 10.1016/j.micpath.2023.106182, 37263448. 37263448

[65] Sharma A. , Gupta V. K. , and Pathania R. , Efflux Pump Inhibitors for Bacterial Pathogens: From Bench to Bedside, Indian Journal of Medical Research. (2019) 149, no. 2, 129–145, 10.4103/ijmr.IJMR_2079_17, 31219077. 31219077 PMC6563736

[66] Grari O. , Ezrari S. , el Yandouzi I. , Benaissa E. , Ben Lahlou Y. , Lahmer M. , Saddari A. , Elouennass M. , and Maleb A. , A Comprehensive Review on Biofilm-Associated Infections: Mechanisms, Diagnostic Challenges, and Innovative Therapeutic Strategies, Microbe. (2025) 8, 100436, 10.1016/j.microb.2025.100436.

